# Thermal Energy Storage of R1234yf, R1234ze, R134a and R32/MOF-74 Nanofluids: A Molecular Simulation Study

**DOI:** 10.3390/ma11071164

**Published:** 2018-07-08

**Authors:** Jieyao Hu, Chao Liu, Lang Liu, Qibin Li

**Affiliations:** 1 Key Laboratory of Low-grade Energy Utilization Technology & System, Ministry of Education, College of Power Engineering, Chongqing University, Chongqing 400044, China; 20161002010t@cqu.edu.cn (J.H.); liuchao@cqu.edu.cn (C.L.); 2 School of Chemical Engineering, The University of Queensland, Brisbane, QLD 4072, Australia; l.liu8@uq.edu.au; 3 Chongqing Key Laboratory of Heterogeneous Material Mechanics, College of Aerospace Engineering, Chongqing University, Chongqing 400044, China

**Keywords:** energy storage, molecular simulation, adsorption, refrigerant, metal organic framework

## Abstract

Thermal energy storage can be carried out by working fluid adsorbing and desorbing in porous materials. In this paper, the energy storage properties of four refrigerants, R1234yf, R1234ze, R134a and R32, with M-metal organic framework (MOF)-74 (M = Zn, Ni, Mg, Co) nanoparticles are investigated using molecular dynamics simulations and grand canonical Monte Carlo simulations. The results show that M-MOF-74 can adsorb more R32 and R134a than R1234yf and R1234ze, as the molecular structures of R32 and R134a are smaller than those of R1234yf and R1234ze. Mg-MOF-74 owns a higher adsorbability than the other MOFs. The energy storage properties of the studied refrigerants can be enhanced when the sum of thermodynamic energy change of MOF particles and the desorption heat of fluid in MOFs is larger than the enthalpy change of pure organic fluid. The R1234yf/M-MOF-74 (M = Co, Mg, Ni) nanofluid can store more energy than other refrigerants/M-MOF-74 (M = Co, Mg, Ni) nanofluid. The energy storage enhancement ratios of R1234yf, R1234ze and R134a with Mg-MOF-74 nanoparticles are higher than those of other M-MOF-74 (M = Co, Ni, Zn) materials.

## 1. Introduction

The energy crisis and environmental pollution are serious issues in human society, as a consequence of the devastating consumption of fossil fuels such as coal, oil and natural gas. In China, the waste heat generated in industries is equivalent to the combustion energy of 340 million tons of standard coal each year. Finding an efficient way to recover the low-grade energy, such as industrial waste heat, geothermal and solar energy, is, hence, essential to combat these issues [1].

The organic Rankin cycle (ORC) uses an organic refrigerant as a working fluid to recover the heat from low-grade energy resources and turns it into electricity, providing a potential way to use the low-grade energy [2]. Further, the efficiency of ORC in low-grade energy recovery could be remarkably enhanced by increasing the thermal properties of the working fluid. McGrail et al. [3] observed reversible adsorption of R123 molecules in metal organic framework (MOF) nanoparticles. They showed that adding MOF nanoparticles into refrigerant (also known as metal-organic heat carrier nanofluids (MOHCs)) could significantly improve the heat capacity of the working fluid and subsequently enhance the efficiency of ORC in recovering the low-grade energy. During the evaporation process in ORC, excess heat is absorbed by MOHCs from the heat resource for the desorption of adsorbate molecules from MOF particles. Further, excess heat is released by the MOHCs during the condensation process as a consequence of the exothermic adsorption of refrigerant molecules into MOF particles. Additionally, the thermal conductivity of the working fluid is enhanced by adding nanoparticles [4]. The reported research works [5,6] indicated that the enhancement of the thermal properties of nanofluid are mainly due to the effective medium theory. Besides, the Brownian motion, layering phenomenon, clustering, ballistic phonon motion, thermal boundary resistance and mass difference scattering will also impact the thermal properties of nanofluid [7,8].

MOFs are highly crystallized by metal ions or metal clusters and organic ligands through the coordination bond or intermolecular interactions, possessing good thermal stability, high specific surface area and strong adsorption affinity for adsorbates. The adsorptive performance of MOFs is found superior to the traditional nanoporous materials such as zeolites and activated carbons [9], making them a promising material for energy storage and gas separation [10,11]. Among a wide range of MOF structures, MOF-74 owns unique secondary building units that have a strong fault tolerance of different metal particles [12]. Nevertheless, its adsorptive performance for organic refrigerates is little understood [13]. Thus, M-MOF-74 (M = Zn, Ni, Mg, Co) is employed as the adsorbent added in the organic refrigerants in this work.

Hitherto, the development of refrigerants has evolved for four generations, with the third and fourth generation of refrigerants being most widely used in practice. In resemblance to the thermodynamic properties of R410a, R32 (difluoromethane, also known as HFC-32), a halogenated hydrocarbon, is treated as an ideal substitute for the second generation ozone-depleting substances [14]. In addition, R134a (tetrafluoroethane, also known as HFC-134a) with zero ODP (ozone depletion potential) is widely used in industry for its excellent cooling performance [15]. Unlike the third generation refrigerants, R32 and R134a, the fourth generation refrigerants are sustainable and environmental friendly, which are proposed to reduce the emission of fluorinated greenhouse gases [16]. Among the hydrofluoroolefins (HFOs) refrigerants, R1234ze (1,3,3,3-tetrafluoropropene, also known as HFO-1234ze) and R1234yf (2,3,3,3-tetrafluoropropene, also known as HFO-1234yf) have demonstrated to be the best working substances to replace the third generation refrigerant, R134a, for their zero ODP, low GWP (global warming potential) and short residence time in the atmosphere [17]. Meanwhile, it has been widely recognized that both R1234yf and R1234ze are promising working fluids in the refrigeration cycle, heat pump and ORC [18,19]. 

For comparative purposes, the adsorption and desorption heats of the third and fourth generation refrigerant molecules, R32, R134a, R1234yf and R1234ze, in MOF-74 were measured by using grand canonical Monte Carlo (GCMC) simulations. In this connection, the maximized heat capacity among the four different nanofluidic fluids comprising the organic refrigerant (R32, R1234yf, R1234ze and R134a) and the MOF-74 particles is unveiled with the aid of molecular dynamics (MD) simulations [20,21,22].

## 2. Materials and Methods

The thermal energy stored in MOHCs can be calculated following [3,23],

(1) $$\Delta h_{\mathrm{MOHCs}}=(1-x)\Delta h_{\mathrm{Fluid}}+x(\int_{T_{0}}^{T_{1}}C_{p}dT){}_{\mathrm{MOFs}}+x\Delta h_{\mathrm{desorption}}$$

or be written as,

(2) $$\Delta h_{\mathrm{MOHCs}}=\Delta h_{\mathrm{Fluid}}+x((\int_{T_{0}}^{T_{1}}C_{p}dT){}_{\mathrm{MOFs}}+\Delta h_{\mathrm{desorption}}-\Delta h_{\mathrm{Fluid}}$$

where the enthalpy gain of MOHCs (Δh_MOHCs_) consists of the enthalpy change of pure organic fluid (Δh_Fluid_), the energy change of the MOF nanoparticles (($\int_{T_{0}}^{T_{1}}C_{p}dT$)_MOFs_) and the enthalpy of desorption (Δh_desorption_) of the refrigerant molecules for the temperature difference of *T*_1_ − *T*_0_. Here, *T*_1_ and *T*_0_ are the temperatures of the heating and cooling resources. *C_p_* is the heat capacity of MOF particles, and x is the mass fraction of MOF particles in MOHCs. It can be concluded that the MOHCs are able to store more thermal energy than the pure fluid when the sum of thermodynamic energy change of MOF particles and desorption heat of fluid in MOFs are larger than the enthalpy change of pure organic fluid. In other words, as long as the second term in the right side of Equation (2) is positive, the energy storage in MOHCs is enhanced compared to that in the pure organic fluid. 

Since the enthalpy of pure species, h_Fluid_ (R32, R1234yf, R1234ze and R134a), is available in NIST [24], the first term on the right side of Equation (2), Δh_Fluid_, can be determined as Δh_Fluid_ = h_Fluid.*T*_ − h_Fluid.*T0*_, where *T*_0_ and *T* are the reference and interested temperatures of the working fluid. Meanwhile, the heat capacity of MOF, C_p_, was measured in our equilibrium molecular dynamic (EMD) simulations. The last part, Δh_desorption_, was calculated as the isosteric heat of adsorption in our GCMC simulations. Subsequently, Δh_MOHCs_ can be readily calculated. 

The EMD and GCMC simulations were performed by using Materials Studio [25]. The COMPASS force field [26] was adopted to describe the intra- and inter-molecular interactions. The Ewald summation method [27] was employed to correct the long-range Coulomb interactions.

### 2.1. MD Computational Details

**Figure 1:**
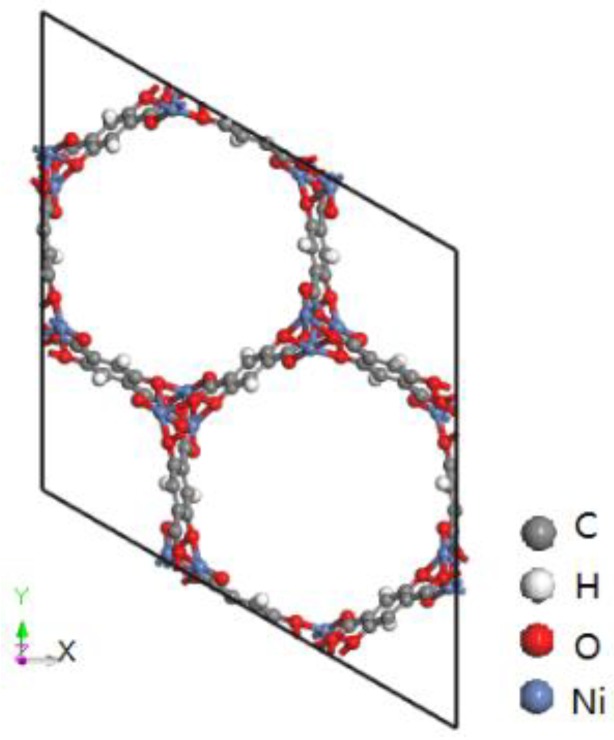
Schematic view of the 1 × 1 × 1 unit cell of metal organic framework MOF-74 (M = Ni).

M-MOF-74 is formed by divalent metal ions (Zn, Mg, Co, Ni, etc.) connected with ligand 2, 5-dihydroxy terephthalic acid. It forms a 2-dimensional six-party channel and a 3-dimensional space network structure like a honeycomb, and metal atoms are octahedrally coordinated (dominated by five oxygen atoms and a water molecule) [28]. The molecular structure of MOF-74 (M = Ni) employed in this work is depicted in Figure 1. The MOF-74 sample comprised of 1 × 1 × 4 unit cells, with 648 atoms (including 288 carbon atoms, 72 oxygen atoms, 216 hydrogen atoms and 72 Ni atoms) was used in our GCMC and EMD simulations. The lattice parameters for the MOF-74 structure are a = b = 25.7856 Å, c = 27.0804 Å, α = β = 90° and γ = 120°.

**Figure 2:**
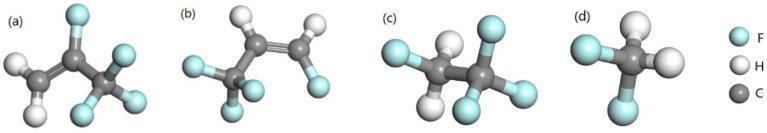
Atomic configurations of R1234yf (**a**); R1234ze (**b**); R134a (**c**) and R32 (**d**).

**Figure 3:**
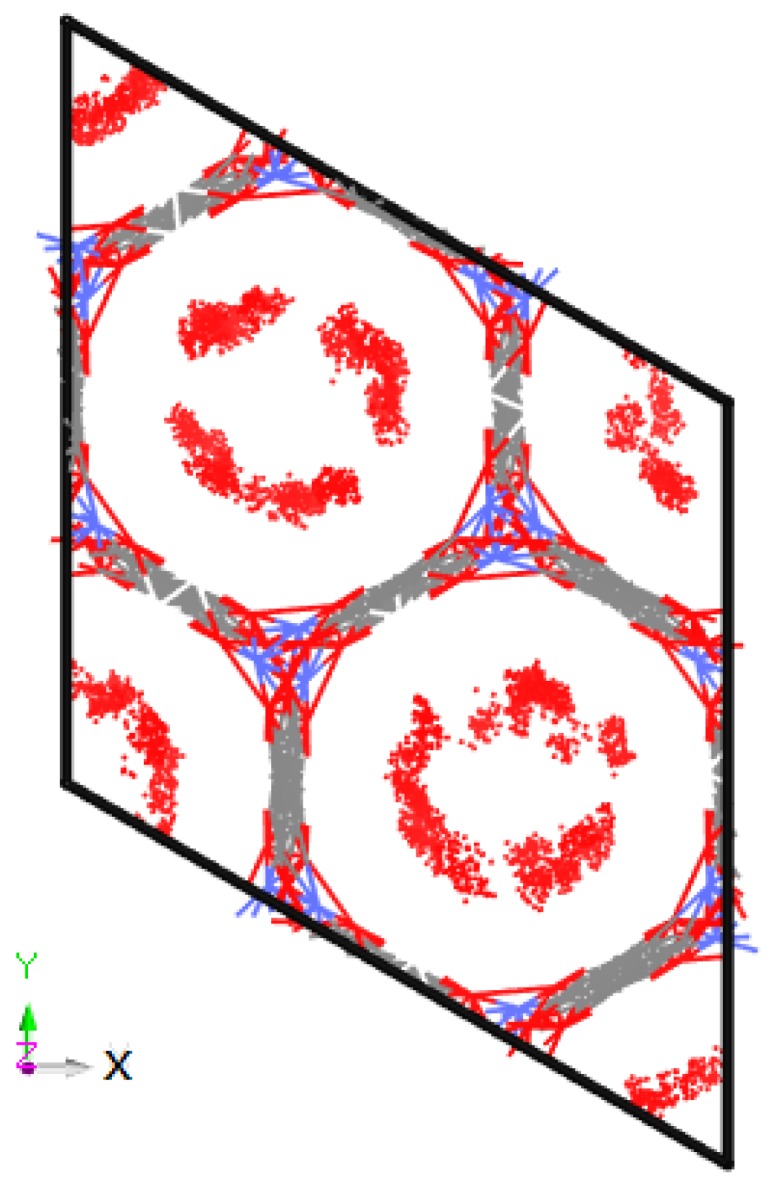
Adsorption sites of R1234ze in Co-MOF-74 at 353 K and 5 MPa.

The molecular configurations of refrigerants R1234yf, R1234ze, R134a and R32 are presented in Figure 2. In addition, the distribution of the adsorption sites of R1234ze in Co-MOF-74 is shown in Figure 3, which was obtained at 353 K and 5 MPa.

In EMD simulations, the thermodynamic energies of M-MOF-74 nanoparticles at different temperatures (293 K, 313 K, 333 K, 353 K, 373 K and 393 K) were computed in canonical (NVT) ensemble via the Forcite module in Materials Studio. The time step was set as 1 fs, with a 200-ps run being used to equilibrate the MOF structures. A 500-ps run after equilibration was further conducted to obtain the thermodynamic energy. The assignment of charges in MOF structures is based on the COMPASS force field, keeping the structures electronically neutral. The Berendsen thermostat was employed to adjust the temperature of the system. The cut-off distance is 12.5 Å. Periodic boundary conditions were applied in the *X*, *Y* and *Z* directions in all of our GCMC and EMD simulations. 

### 2.2. GCMC Computational Details

The GCMC simulations are able to calculate the adsorption isotherms (293 K, 313 K, 333 K, 353 K, 373 K and 393 K) of refrigerants (R1234yf, R1234ze, R134a and R32) in the M-MOF-74 nanoparticles via the Sorption module in Materials Studio. The pressure range of 1–10,000 kPa is investigated in this work. The fugacity is calculated by the Peng–Robinson equation. For each point of the adsorption isotherm, the equilibration time was 10,000 cycles with another 100,000 cycles for statistics. Here, the simulation runs 5,500,000 cycles to obtain the adsorption isotherm at each temperature (293 K, 313 K, 333 K, 353 K, 373 K and 393 K).

## 3. Results and Discussion

### 3.1. Thermodynamic Energy of MOF-74

**Figure 4:**
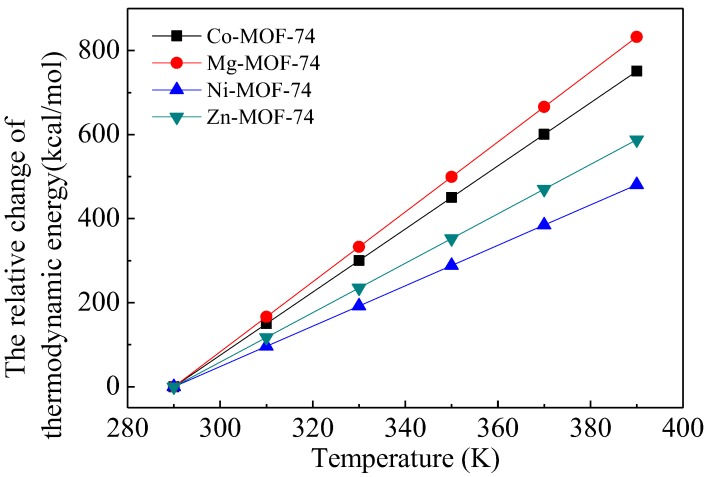
Variation of the relative change of the thermodynamic energy of M-MOF-74 with temperature, with M denoting Co, Mg, Ni and Zn.

Our previous work [29] indicated that the present method is an efficient approach to obtain the thermodynamic parameters of MOFs. The changes of the thermodynamic energies of M-MOF-74 structures at different temperatures relative to that at the reference temperature of 293 K are plotted in Figure 4. The thermodynamic energy of M-MOF-74 increases linearly with temperature. The increment of thermodynamic energy per unit temperature is the heat capacity of MOFs, *C_p_*, i.e., *ρC_p_*_-Co-MOF-74_ = 3.34 (J/cm^3^·K), *ρC_p_*_-Mg-MOF-74_ = 3.74 (J/cm^3^·K), *ρC_p_*_-Ni-MOF-74_ = 2.14 (J/cm^3^·K), *ρC_p_*_-Zn-MOF-74_ = 2.62 (J/cm^3^·K). These values are larger than *ρC_p_*_-MOF-5_ = 1.24 (J/cm^3^·K), which was calculated in our previous work [29]. The difference of heat capacities between MOF-74 and other MOFs was expected to be a result of the difference in their structures and components [3,30].

### 3.2. Adsorption Isotherms

**Figure 5:**
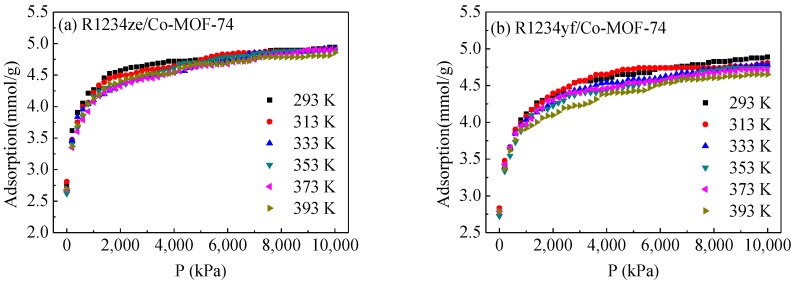

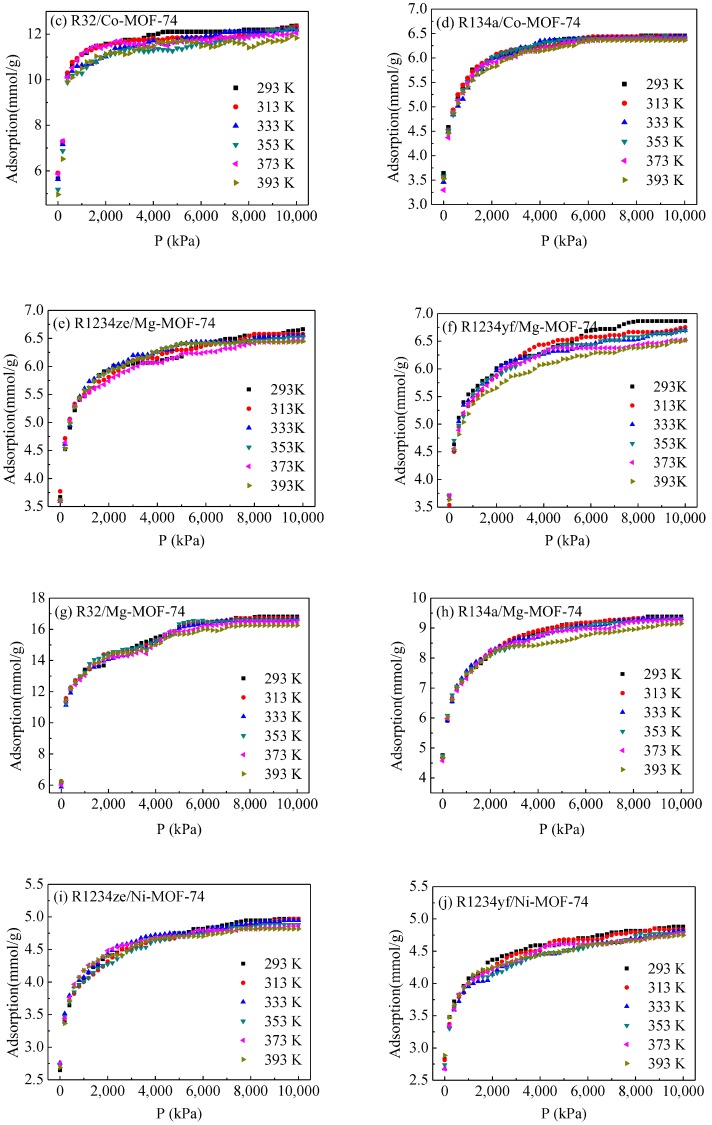

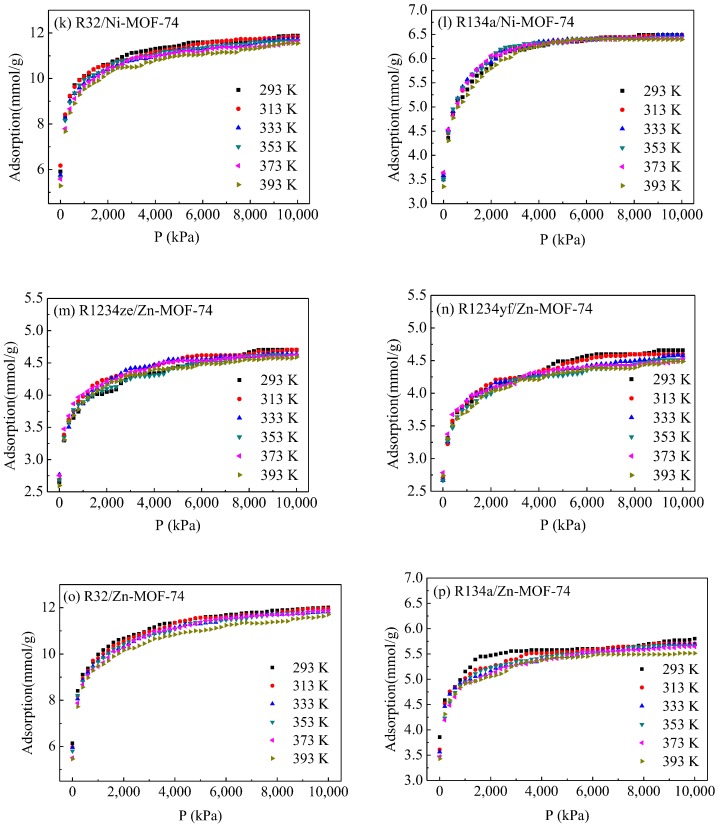
Adsorption isotherms of refrigerants in M-MOF-74 at different temperatures. (**a**) R1234ze/Co-MOF-74; (**b**) R1234yf/Co-MOF-74; (**c**) R32/Co-MOF-74; (**d**) R134a/Co-MOF-74; (**e**) R1234ze/Mg-MOF-74; (**f**) R1234yf/Mg-MOF-74; (**g**) R32/Mg-MOF-74; (**h**) R134a/Mg-MOF-74; (**i**) R1234ze/Ni-MOF-74; (**j**) R1234yf/Ni-MOF-74; (**k**) R32/Ni-MOF-74; (**l**) R134a/Ni-MOF-74; (**m**) R1234ze/Zn-MOF-74; (**n**) R1234yf/Zn-MOF-74; (**o**) R32/Zn-MOF-74; (**p**) R134a/Zn-MOF-74.

Figure 5 depicts the adsorption isotherms of refrigerants including R1234yf, R1234ze, R134a and R32 in M-MOF-74 at different temperatures, in which symbols represent the results measured in our GCMC simulations. Among the four refrigerants investigated, R32 was demonstrated to have the highest adsorption capacity in the M-MOF-74 within the temperature range considered, evident in Figure 5c,g,k,o, followed by R134a. This was due to the molecule structure of refrigerants. As is shown in Figure 2, the R32 molecule has a structure with a smaller effective diameter. R1234yf and R1234ze own structures with large effective diameters. At the high pressures, when the adsorption approached saturation, the adsorbate molecules experienced a strong repulsive force from the tightly-packed neighbouring molecules and the host structure [31,32]. This repulsive interaction should be further enhanced for the molecules having a larger size, leading to reduction of the adsorption volume available in MOF-74 for R1234yf, R1234ze and R134a compared to R32. Consequently, the highest adsorption capacity was achieved for R32 in the pores of MOF-74. On the other hand, the Mg-MOF-74 adsorbed more refrigerants than other MOF-74s. This is because the ionic radius of Mg^2+^ is smaller than those of Co^2+^, Ni^2+^ and Zn^2+^, which results in a larger adsorption volume in the Mg-MOF-74. More importantly, the interactions between Mg^2+^ and adsorbate molecules were stronger than the counterpart interactions for Co^2+^, Ni^2+^ and Zn^2+^ ions [33]. The enthalpy of desorption calculated by GCMC simulations is shown in Appendix A, confirming this statement. 

### 3.3. Thermal Energy Storage Properties of MOHCs

**Figure 6:**
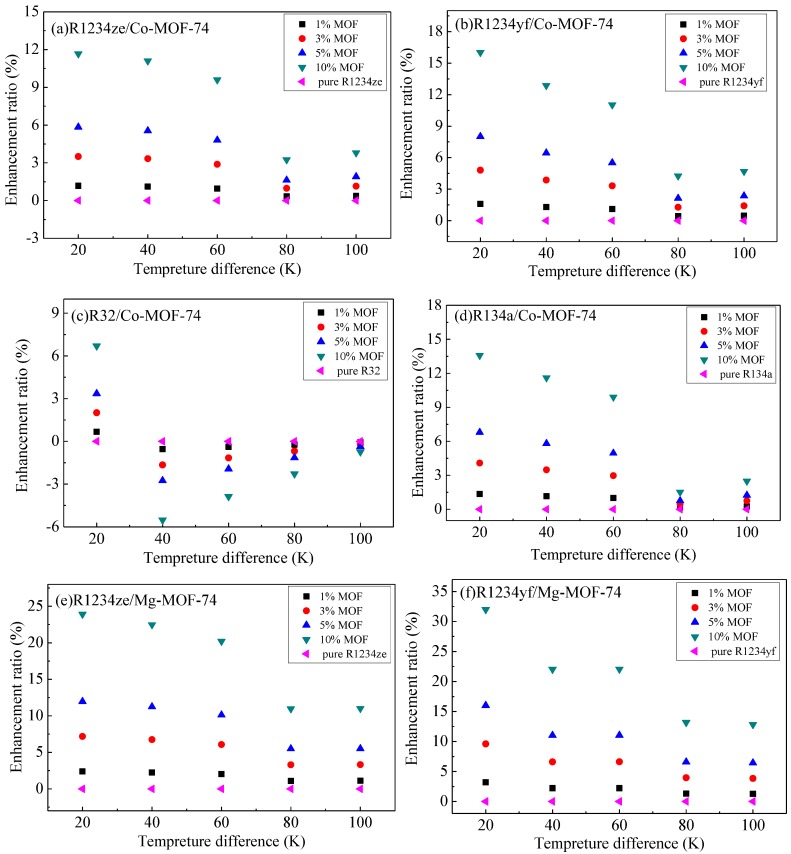

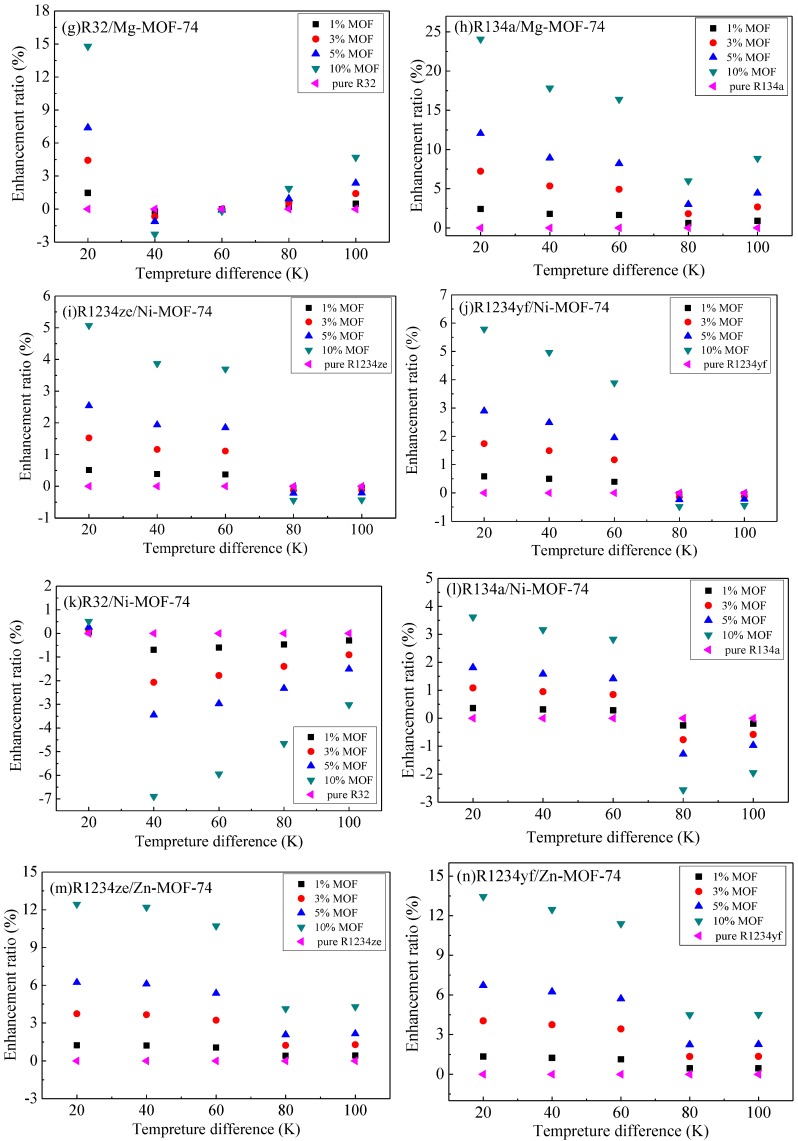

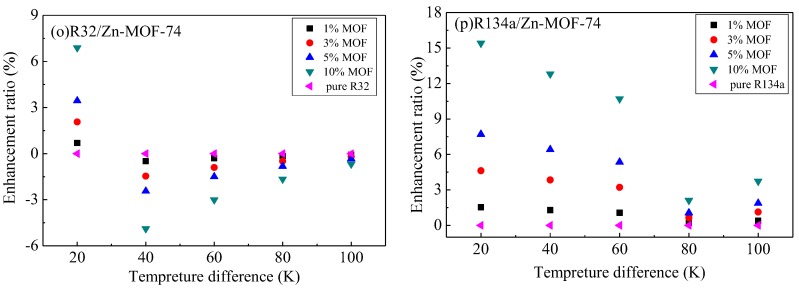
The relationship between the thermal energy storage and temperature difference of the adsorbates (R1234yf, R1234ze, R134a, R32)/MOF-74 mixture at 3500 kPa. *(***a**) R1234ze/Co-MOF-74; (**b**) R1234yf/Co-MOF-74; (**c**) R32/Co-MOF-74; (**d**) R134a/Co-MOF-74; (**e**) R1234ze/Mg-MOF-74; (**f**) R1234yf/Mg-MOF-74; (**g**) R32/Mg-MOF-74; (**h**) R134a/Mg-MOF-74; (**i**) R1234ze/Ni-MOF-74; (**j**) R1234yf/Ni-MOF-74; (**k**) R32/Ni-MOF-74; (**l**) R134a/Ni-MOF-74; (**m**) R1234ze/Zn-MOF-74; (**n**) R1234yf/Zn-MOF-74; (**o**) R32/Zn-MOF-74; (**p**) R134a/Zn-MOF-74.

**Figure 7:**
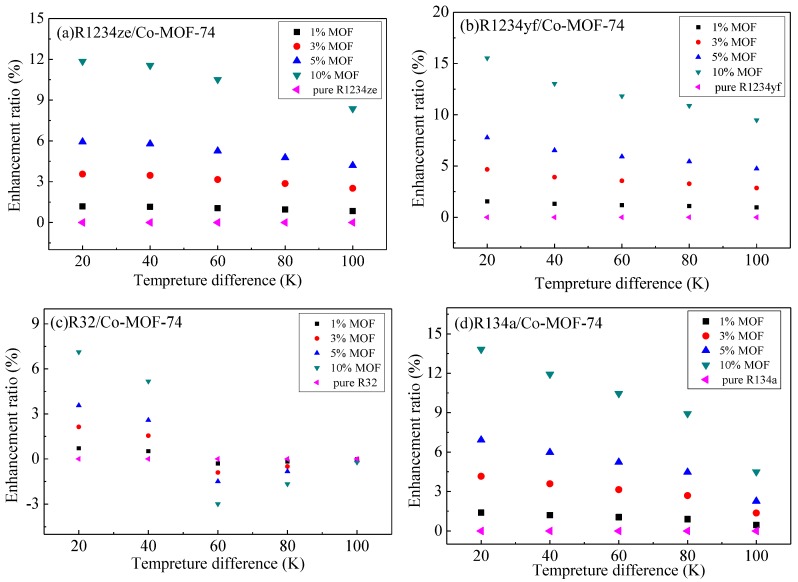

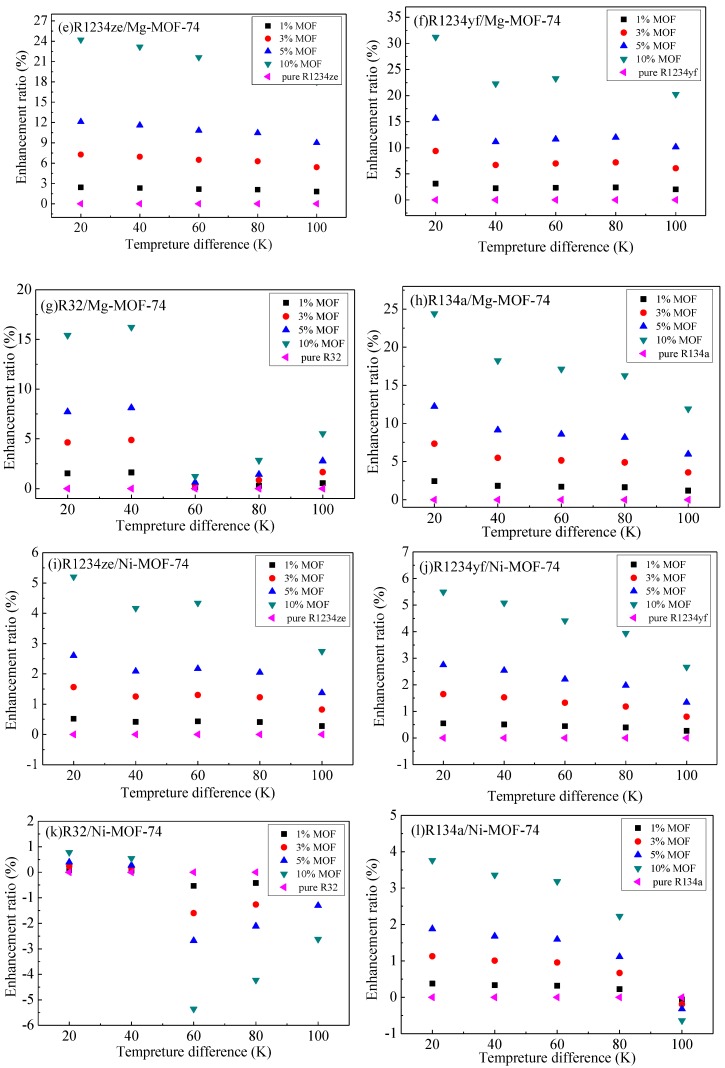

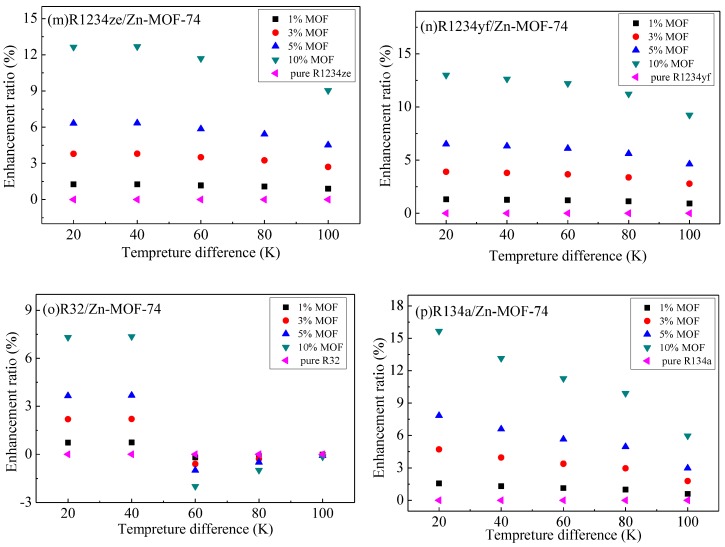
The relationship between the thermal energy storage and temperature difference of the adsorbates (R1234yf, R1234ze, R134a, R32)/MOF-74 mixture at 5000 kPa. (**a**) R1234ze/Co-MOF-74; (**b**) R1234yf/Co-MOF-74; (**c**) R32/Co-MOF-74; (**d**) R134a/Co-MOF-74; (**e**) R1234ze/Mg-MOF-74; (**f**) R1234yf/Mg-MOF-74; (**g**) R32/Mg-MOF-74; (**h**) R134a/Mg-MOF-74; (**i**) R1234ze/Ni-MOF-74; (**j**) R1234yf/Ni-MOF-74; (**k**) R32/Ni-MOF-74; (**l**) R134a/Ni-MOF-74; (**m**) R1234ze/Zn-MOF-74; (**n**) R1234yf/Zn-MOF-74; (**o**) R32/Zn-MOF-74; (**p**) R134a/Zn-MOF-74.

As previously mentioned, the thermal energy storage in MOHCs can be calculated according to Equation (1) or Equation (2). The relationships between thermal energy storage and temperature difference (the temperature of the cold source is 293 K) of the adsorbates (R1234yf, R1234ze, R134a, R32)/MOF-74 mixture at 3500 and 5000 kPa are shown in Figure 6 and Figure 7, respectively. The thermal energy storage capacity of adsorbates (R1234yf, R1234ze, R134a, R32) increased with the mass ratio of MOF-74 nanoparticles in the working fluid. The percentage enhancement of the thermal energy was calculated as [Δh_MOHCs_(ΔT) − Δh_Fluid_(ΔT)]/Δh_Fluid_(ΔT), where ΔT is the temperature difference relative to the reference temperature of 293 K. 

In the M-MOF-74 (M = Co, Mg, Ni) structures, the energy storage enhancement ratio of R1234yf was higher than that of the other three adsorbates. However, in Zn-MOF-74, R134a achieved the highest energy storage enhancement ratio among the four refrigerants considered. The energy storage enhancement ratio of R32 in Zn-MOF-74 was higher than that of the other three M-MOF-74 (M = Co, Mg, Ni) structures, while the energy storage enhancement ratios of R1234yf, R1234ze and R134a in Mg-MOF-74 were higher than those of the other three M-MOF-74 (M = Co, Ni, Zn). It is worth noting that the energy storage enhancement ratio at 5000 kPa is higher than that of 3500 kPa. However, the negative enhancement of thermal energy storage properties is found in a certain temperature range in Figure 6c,g,i,j,k,l,o and Figure 7c,k,l,o. This is because the enthalpy difference of the refrigerant was larger than the sum of the thermodynamic energy change of M-MOF-74 and desorption heat at the corresponding temperature range.

## 4. Conclusions

In this work, molecular simulations including MD and GCMC simulations were employed to investigate the energy storage by the adsorption of adsorbates (R1234yf, R1234ze, R134a, R32) in MOF-74. From the results, the following conclusions could be drawn.

Among the four different refrigerants, R32 has the largest adsorption capacity in M-MOF-74 materials, followed by R134a. This is due to the molecular structures of R32 and R134a being smaller than those of R1234yf and R1234ze. Mg-MOF-74 adsorbs more refrigerant than the other three MOFs, as the adsorption interaction between Mg-MOF-74 and refrigerants is stronger than the other MOFs.

The energy storage enhancement ratio of R1234yf with M-MOF-74 (M = Co, Mg, Ni) nanoparticles was higher than other refrigerants with M-MOF-74 (M = Co, Mg, Ni) nanoparticles. The energy storage enhancement ratio of R134a with Zn-MOF-74 nanoparticles was enhanced compared to other refrigerants with Zn-MOF-74 nanoparticles; while the energy storage enhancement ratio of R32 in Zn-MOF-74 was higher than those of the other three M-MOF-74 (M = Co, Mg, Ni) structures. The energy storage enhancement ratios of R1234yf, R1234ze and R134a in Mg-MOF-74 were higher than those of the other three M-MOF-74 (M = Co, Ni, Zn). Besides, the negative enhancement of thermal energy storage properties is found under certain conditions of MOHCs.

## Abbreviations

Δh_MOHCs_	The enthalpy change of MOHCs (kJ/kg)
Δh_Fluid_	The enthalpy change of pure organic fluid (kJ/kg)
Δh_desorption_	The enthalpy of desorption (kJ/mol)
*T*	Temperature (K)
*C_p_*	The heat capacity of MOFs (kJ/(mol·K))
x	The mass fraction of MOFs particle in MOHCs
*ρ*	Density (g/cm^3^)

## Appendix A. Enthalpy of Desorption Calculated by GCMC Simulations

**Table A1:** The adsorption heat calculated by grand canonical Monte Carlo (GCMC) simulations (kJ/mol).

MOHCs	293 K_Sim_	313 K_Sim_	333 K_Sim_	353 K_Sim_	373 K_Sim_	393 K_Sim_
R1234ze/Co	128.19	127.90	127.03	127.26	127.10	126.84
R1234yf/Co	117.61	116.50	116.47	115.96	115.47	114.57
R32/Co	75.67	75.20	73.86	75.39	73.66	74.39
R134a/Co	124.93	123.61	123.31	123.14	123.03	122.40
R1234ze/Mg	279.73	279.42	278.58	278.25	277.57	276.74
R1234yf/Mg	235.72	234.14	237.97	233.39	230.97	230.39
R32/Mg	177.06	176.96	174.94	173.94	173.61	171.57
R134a/Mg	257.38	256.32	258.36	259.78	258.09	256.47
R1234ze/Ni	127.00	126.54	126.26	125.93	125.02	124.87
R1234yf/Ni	116.32	115.67	115.37	115.01	114.14	113.70
R32/Ni	76.00	75.89	75.64	75.59	74.41	71.17
R134a/Ni	123.42	123.42	123.09	122.68	122.48	122.20
R1234ze/Zn	128.18	127.67	127.28	126.82	126.11	125.84
R1234yf/Zn	117.87	117.37	117.21	116.61	115.99	115.39
R32/Zn	116.89	116.58	116.27	114.68	114.06	115.62
R134a/Zn	155.11	153.91	153.90	153.87	153.45	152.49
